# Developing an Electronic Handover System for On-Call Doctors in a South London Mental Health Trust

**DOI:** 10.1192/bjo.2024.335

**Published:** 2024-08-01

**Authors:** Ahmed Abdelsamie, Laith Alexander, Rebecca Wilkinson, Olivia Dawson, Prateek Yadav

**Affiliations:** South London and Maudsley NHS Foundation Trust, London, United Kingdom

## Abstract

**Aims:**

To replace pre-existing paper-based and informal handover systems with a confidential electronic handover system for on-call doctors across a large South London mental health trust, thereby improving the safety and quality of handovers.

**Methods:**

A quality improvement project was registered within our trust. At baseline, we surveyed core trainees, GP trainees, and locum doctors about their experiences using a paper handover system for on-calls at the Bethlem Royal Hospital and Lambeth Hospital, South London and the Maudsley NHS Foundation Trust (SLaM). Their feedback guided the implantation of a confidential and secure electronic handover system integrated into the trust's Microsoft SharePoint, using the *Microsoft To Do* app and then *Microsoft Teams*. We alerted doctors to these changes via formal and informal means, such as trust inductions, emails and through communication with trust junior doctor representatives. After a period of 8 and 24 weeks, we assessed the initiative's success by collecting both qualitative and quantitative data from on-call doctors about their experience with the handover system. Based on feedback, we made multiple adjustments to improve the system, which was later adopted at Lambeth Hospital. The *Microsoft To Do* app was then replaced by a channel on *Microsoft Teams* to ensure wider access.

**Results:**

15 doctors responded to the baseline survey. Handover practices were varied and included paper-based handovers, phone calls, and emails. Mean doctors’ ratings for the pre-existing handover systems were 3.2/5 for overall quality (1: very poor; 5: very good) and 2.7/5 for safety (1: very unsafe; 5: very safe). 60% (n = 9) of doctors said tasks would sometimes be missed in the pre-existing handover system. 21 doctors responded to 2 post-change surveys. Mean doctors’ ratings of overall quality were 4.6/5 and safety were 4.5/5. Qualitative feedback highlighted that a verbal handover was still necessary to complement the electronic system, and that locum doctors would need to have access to the system as well as consultants and registrars during periods of industrial action.

**Conclusion:**

An electronic handover system was successfully implemented to replace a predominantly paper-based handover system at two large mental health hospitals in South London, and on-call doctors reported improvements in handover safety and handover quality. Future work aims to implement a consistent electronic handover system across other hospitals in SLaM and other trusts and transition fully to *Microsoft Teams* for broader accessibility.

